# Electronic materials with a wide band gap: recent developments

**DOI:** 10.1107/S2052252514017229

**Published:** 2014-08-29

**Authors:** Detlef Klimm

**Affiliations:** a Leibniz Institute for Crystal Growth, Max-Born-Straße 2, 12489 Berlin, Germany

**Keywords:** electronic materials, wide band gap materials, semiconductors, silicon, germanium

## Abstract

The development of semiconductor electronics is reviewed briefly, beginning with the development of germanium devices (band gap *E*_g_ = 0.66 eV) after World War II. A tendency towards alternative materials with wider band gaps quickly became apparent, starting with silicon (*E*_g_ = 1.12 eV). This improved the signal-to-noise ratio for classical electronic applications. Both semiconductors have a tetrahedral coordination, and by isoelectronic alternative replacement of Ge or Si with carbon or various anions and cations, other semiconductors with wider *E*_g_ were obtained. These are transparent to visible light and belong to the group of wide band gap semiconductors. Nowadays, some nitrides, especially GaN and AlN, are the most important materials for optical emission in the ultraviolet and blue regions. Oxide crystals, such as ZnO and β-Ga_2_O_3_, offer similarly good electronic properties but still suffer from significant difficulties in obtaining stable and technologically adequate *p*-type conductivity.

## Abstract 2

Usually, semiconductors with a band gap *E*_g_ ≃ 3 eV or larger are called wide band gap materials. Their optical emission can span the whole of the visible spectrum, enabling the development of devices for solid-state lighting. In addition, a large *E*_g_ results in a high electrical breakthrough field, which is interesting for high-power electronics.

## 1. Introduction   

Semiconductors are crystalline or amorphous substances with a full valence band and an empty conduction band. These two bands are separated by the band gap energy *E*_g_. Electronic charge transport in such systems is possible by fulfilling the following conditions:

(i) Electrons must be emitted from the valence to the conduction band, *e.g.* by thermal emission. ‘Intrinsic conduction’ then results from the movement of these negative free electrons and the corresponding positive ‘defect electrons’ (or holes) in opposite directions, if an electric field is applied. The conduction rises with temperature *T* and becomes significant if the average thermal energy of the electrons *k*_B_ *T* (∼25 meV at room temperature; *k*_B_ is the Boltzmann constant, 1.3806488 × 10^−23^ m^2^ kg s^−2^ K^−1^) approaches *E*_g_/2. At sufficiently high *T*, this condition is fulfilled by every material.

(ii) Small amounts of suitable impurities can create additional ‘dopant’ levels in the otherwise empty band gap. ‘Shallow acceptor’ levels are situated close to the bottom of the gap, typically a few tens of meV above the valence band. Consequently, at room temperature they are filled almost completely by thermal emission, leaving behind holes in the valence band (*p*-type conductivity). For ‘shallow donors’ close to the top of the gap the situation is opposite: these levels can emit electrons into the conduction band (*n*-type conductivity). Deep acceptors or deep donors, which are situated close to the middle of the energy gap, do not contribute significantly to the electric carrier concentration, and thus not to the electrical conductivity.

Insulators are materials with very large *E*_g_, typically in excess of 3–5 eV. However, this limit is quite arbitrary and turns out to be subject to technological developments: substances such as aluminium nitride or even diamond are nowadays usually considered to be semiconductors. Semiconductors with *E*_g_ considerably larger than the ‘normal’ semiconductors Si, Ge or GaAs (see Tables 1 ▶ and 3) are called wide band gap semiconductors, and are the topic of this article. In contrast, narrow band gap semiconductors have a small *E*_g_ of a few hundreds of  meV.

## 2. A short look at history: germanium and silicon   

Faraday revealed as early as the 1830s that some substances show an increase in their electrical conductivity with *T*, which is in contrast with metals. However, it took more than a century before semiconducting Ge crystals were grown with the Czochralski method (Teal & Little, 1950 ▶; Teal *et al.*, 1951 ▶; Uecker, 2014 ▶), which paved the way to the broad technological relevance of semiconductors. The growth of germanium bulk crystals with high crystalline perfection was a breakthrough and became the origin of today’s semiconductor-based electronic industry. Unfortunately, *E*_g_ is comparatively narrow for germanium, leading to a particularly large intrinsic conduction which cannot be controlled by *p*–*n* junctions, and which is the origin of electronic noise.

Table 1 ▶ summarizes data from several sources (Kasap & Capper, 2007 ▶; GTT Technologies, 2013 ▶; Glusker *et al.*, 1994 ▶) for the main group 4 elements crystallizing in the diamond structure, like germanium. In the table, *a*_0_ is the lattice constant; *T* range means the limit where disintegration of the diamond phase occurs, which for Si and Ge is by melting, for C is by transformation from metastable diamond to thermodynamically stable graphite, and for grey α-Sn is by transformation to tetragonal β-Sn, which is stable under ambient conditions. For every energy gap a value of λ_g_ = *hc*/*E*_g_ can be calculated, which is the minimum optical wavelength for which the material is transparent. Compared with Ge, Si has a much broader *E*_g_, which reduces the intrinsic conduction and electronic noise of devices made from it. Indeed, silicon (mainly Czochralski-grown) is today the most important semiconductor material. Grey tin is not technologically relevant, but diamond receives increasing attention as a truly wide band gap semiconductor. Poly- and single-crystalline diamond are typically grown by chemical vapour deposition (CVD) processes, and devices such as diodes and transistors show excellent breakthrough stability up to as much as 20 MV cm^−1^. It is expected to outperform other wide band gap semiconductors such as 4H-SiC and GaN at 300°C by more than one order of magnitude (Hiraiwa & Kawarada, 2013 ▶; Shiomi & Kumazawa, 1996 ▶).

**Figure 1:**
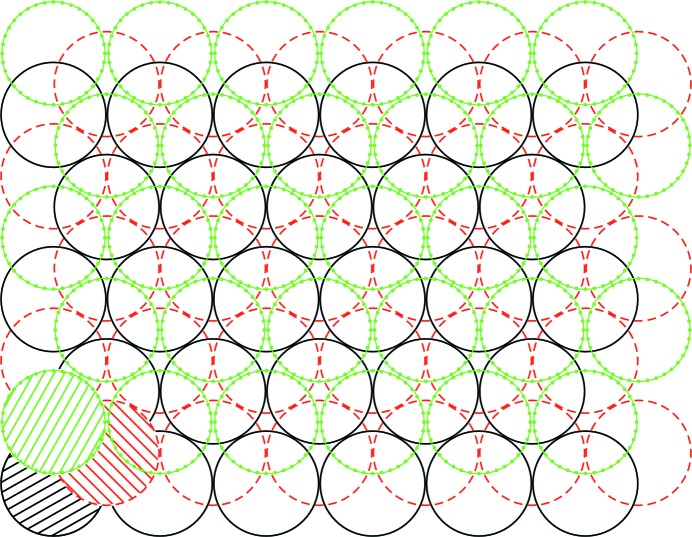
The stacking of layers *A*–*B*–*C* (from bottom to top) in the diamond structure. One atom of each layer is hatched for a better demonstration of the stacking sequence.

The diamond structure is characterized by *sp* ^3^ hybrid orbitals which repel each other and are therefore directed from the central atom to the corners of a regular tetrahedron. The tetrahedra are arranged in layers, and if the position of the first layer (perpendicular to the *c* axis) is designated *A*, subsequent layers are stacked in the somewhat shifted positions *B* and *C*, resulting in a cubic stacking *A*–*B*–*C*–*A*–*B*–*C* (see Fig. 1 ▶). In contrast, the orbitals in the stable modification of carbon, graphite, are *sp* ^2^ hybridized. Here, repulsion directs the orbitals in a planar fashion, 120° apart. The remaining non-hybridized delocalized electron is situated out of the carbon plane and is the origin of the almost metallic conductivity of graphite parallel to the basal (carbon atom) plane of its hexagonal structure. It is notable that carbon can form a wide variety of other allotropes (graphene, nanotubes, buckminster­fullerenes, lonsdaleite), some of which are the subject of intensive research, but they have not yet reached the level of particular technological relevance. In lonsdaleite, the carbon tetrahedra show hexagonal stacking, *A*–*B*–*A*–*B*. In the schematic of Fig. 1 ▶ this means that the uppermost layer is in the same position as the bottom layer.

**Figure 2:**
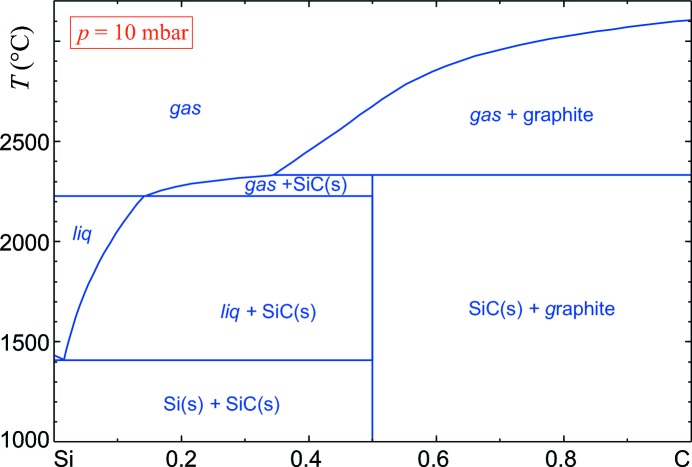
The silicon–carbon phase diagram.

The diamond-type elements of main group 4 show partial (complete only in the case of Si–Ge) mutual solubility. Typically, the solubility is larger (possibly under non-equilibrium conditions) for epitaxial layers. Soref (2014 ▶) discussed the properties of alloys in the C–Si–Ge–Sn quaternary system and claimed that only alloys containing tin might offer direct band gaps. However, this publication disregarded the existence of the only intermediate compound in this system which is an important semiconductor, namely silicon carbide, and it will be discussed in §3. The homogeneity range of the three solid phases in the phase diagram (Fig. 2 ▶) is only a few parts per million.

**Figure 3:**
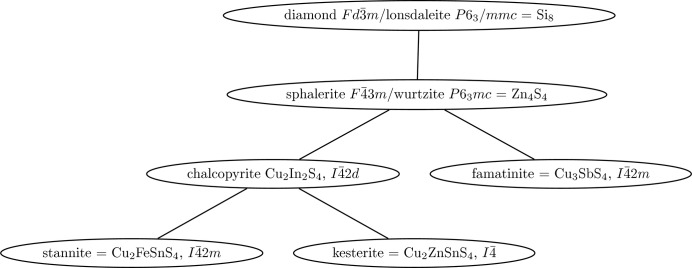
The derivation of tetrahedral multi-cation compounds from element structures.

From the cubic diamond structure and the hexagonal lonsdaleite structure, binary or ternary compound structures, respectively, can be derived if the C atoms are substituted in an ordered manner by other atoms in such a way that the average of four electrons per atomic site is maintained; for an overview, see *e.g.* Parthé (1964 ▶) and Delgado (1998 ▶). The structures of diamond and lonsdaleite, and derived tetrahedrally bound compounds, are called adamantane types. Fig. 3 ▶ shows the interdependency of such tetrahedral structures, with several sulfides as examples. It is obvious that the crystal symmetry drops with increasing chemical complexity. Only a few of these structure types, namely diamond, sphalerite and wurtzite, are found for wide-band gap semiconductors. Some others, such as kesterite and stannite, with narrow *E*_g_ around 1.0–1.5 eV, are technologically relevant, *e.g.* as absorbers for thin-film solar cells (Redinger *et al.*, 2011 ▶).

## 3. Binary compounds derived from diamond and lonsdaleite   

These *AB* compounds comprise alternating *AB*_4_ (or *A*_4_ *B*, respectively) tetrahedra which are linked through their corners. Different stackings for the tetrahedron layers are observed, as for diamond and lonsdaleite. If diamond stacking is performed with the *AB*_4_ tetrahedra, the atom sites are identical to those of diamond itself, with just the *A* and *B* atoms alternating. The structure remains cubic, but the symmetry is lowered to space group 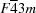. This is the sphalerite (= zincblende) structure type. In a similar way, lonsdaleite stacking of *AB*_4_ tetrahedra also results in conservation of the atomic positions with alternating atom types. The resulting wurtzite structure belongs to space group *P*6_3_ *mc*. In an ideal wurtzite structure (stacking of ideal spheres), one has *c*/*a* = (8/3)^1/2^ = 1.633, but this is not always fulfilled and results then in distorted tetrahedral bonding.

Often the type of stacking is fixed for one specific *AB* compound, because deviations from the ideal stacking increase the lattice energy of the crystal by the stacking-fault energy γ. Although γ is usually given in units of energy per area, scaling in energy per atom is preferred, as a certain number of bonds have to be broken to create the stacking fault. Gottschalk *et al.* (1978 ▶) showed that γ drops almost linearly if the ionicity of the *A*—*B* bonds rises, and for cubic *A* ^III^ *B* ^V^ compounds γ ranges from 53 ± 7 meV atom^−1^ for GaSb to 17 ± 3 meV atom^−1^ for InP. Low γ values are detrimental to crystal growth processes because even small thermal stresses can lead to stacking faults which impede the electronic properties of the material.

The similarity of carbon and silicon is responsible for the low ionicity of SiC. In particular, a huge variety of stacking orders can be observed for this compound, called polytypes. It turns out that different SiC polytypes are energetically almost identical and all of them (especially α-SiC, see below) have very low stacking-fault energies (Hong *et al.*, 2000 ▶). Consequently, they can easily coexist or be transformed into each other, or switching between polytypes can occur during growth (Rost *et al.*, 2005 ▶). The polytypes are described by the Ramsdell notation, which is a number giving the period of the stacking followed by the letter H, C or R, indicating that the stacking symmetry is hexagonal, cubic or rhombohedral, respectively. In fact, SiC can belong to only one of the four space groups *P*3*m*1, *R*3*m*1, *P*6_3_ *mc* or 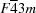 (Krishna & Pandey, 2001 ▶). Historically, cubic (zincblende, 3C) SiC is labelled β-SiC, whereas the other modifications are called α-SiC. Table 2 ▶ reports some relevant SiC polytypes between the pure hexagonal 2H and pure cubic 3C extremes. The second line reports the average thickness of a single layer, which does not differ much. All polytypes have rather large indirect band gaps, especially non-cubic α-SiC.

SiC is mechanically hard, chemically inert, and can be integrated well into standard semiconductor production lines. The growth of single crystals is a challenge, as can be seen readily from the Si–C phase diagram in Fig. 2 ▶ which was calculated for a reduced pressure of *p* = 10 mbar (1 bar = 100 000 Pa). For significantly larger or even ambient *p*, the ‘liq’ phase field for Si-rich compositions extends to higher *T*, which then results in peritectic melting of SiC to an Si-rich melt and solid carbon (graphite). Only under reduced *p* << 1 bar is solid SiC in equilibrium with ‘gas’, enabling sublimation growth (physical vapour transport, PVT) which is the standard growth technique for SiC single crystals. Alternatively, at *T* ≤ 2300°C growth from melt solutions is an option (top-seeded solution growth, TSSG), and this was demonstrated and compared with PVT by Hofmann & Müller (1999 ▶). Often, a metal (Fe, Ni, Cr, Ti or Li) is added to the melt. Different polytypes (*e.g.* 4H) are now commercially available as wafers of 150 mm diameter, with *n*-type and *p*-type doping. The large band gap, good carrier mobility and stability of SiC allow the production of electronic and optoelectronic devices with superior properties and a high breakdown field that are able to work even under harsh conditions. It should be noted that SiC, under the name carborundum, is a mass product used *e.g.* as an abrasive and for specialized ceramics in car brakes. Here, as for electronics, its high thermal conductivity is beneficial as it allows the removal of waste heat.

SiC is the only tetrahedrally bound semiconductor that can be derived from diamond or lonsdaleite by replacing C alternately with C or Si, respectively. If the structure is derived from diamond, one obtains the cubic zincblende (sphalerite) structure; if it is derived from lonsdaleite, the hexagonal wurtzite structure is obtained. It is notable that the names of both structure types are derived from zinc sulfide (ZnS), which can be found as a natural mineral in both structure types. Other isoelectronic replacements, with identical structural features, can be obtained by replacing C (group 4 of the periodic system) alternately with elements from groups 3 and 5. Replacement with elements from main groups 2 and 6 results mainly in compounds with the sodium chloride structure, with a few exceptions such as the insulator BeO (Austerman *et al.*, 1997 ▶) and the wide band gap semiconductor MgTe (Kuhn *et al.*, 1971 ▶) belonging to the wurtzite type. However, many subgroup elements also form bivalent ions: the corresponding Me^2+^ chalcogenides often crystallize in the sphalerite or wurtzite structure and are semiconductors. Some of these *A* ^III^ *B* ^V^ or *A* ^II^ *B* ^VI^ semiconductors with technological relevance are shown in Table 3 ▶; even the *A* ^I^ *B* ^VII^ compound silver iodide crystallizes below ≲162°C in the wurtzite structure and has a wide band gap.

Those compounds with higher ionicity tend to crystallize in the wurtzite structure, and the higher ionicity goes along with a larger *E*_g_. For GaP, the optical transparency reaches the visible range and wafers are transparent to red light. AlP (wider *E*_g_ = 2.45 eV) with the sphalerite structure only has relevance as a semiconductor in mixed crystals with other *A* ^III^ *B* ^V^ compounds. Pure AlP, in contrast with other group 3 phosphides and arsenides, tends to hydrolyze with moisture to form poisonous phosphine gas (PH_3_) and is used as a pesticide. Gallium and indium phosphides, arsenides and, partially, antimonides for semiconductor applications are typically grown as bulk single crystals from the melt, either by crystallization inside a crucible from the bottom to the top (Bridgman; vertical gradient freeze or VGF) or by pulling (Czochralski). Arsenides and more so phosphides tend to have a large arsenic or phosphorus vapour pressure (up to several tens of bar) at their melting points. Disintegration of these semiconductor compounds can be avoided by overpressure and ‘liquid encapsulation’ of the material with B_2_O_3_, which melts at 450°C, significantly lower than the semiconductor, and forms a liquid layer on top of the melt.

**Figure 4:**
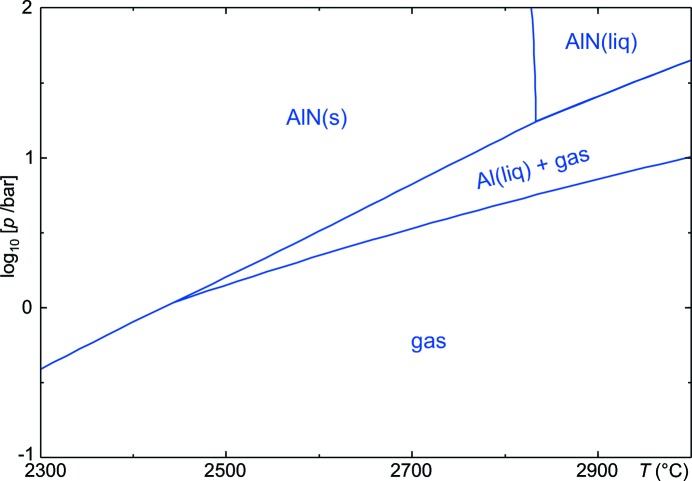
Temperature–pressure phase diagram for AlN, demonstrating the decomposition AlN 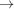 Al + 0.5N_2_ at insufficient pressure. Calculated using *FactSage 6.4*.

Among the group 3 nitrides, BN has not yet reached its full potential. Different modifications occur and for the wurtzite-type *E*_g_ = 5.2 eV is reported, which makes the material almost an insulator. An excellent database on this interesting compound can be found on the World Wide Web (Ioffe Database, 2014 ▶; http://www.ioffe.ru/SVA/NSM/Semicond/BN/index.html). For the other group 3 elements, the affinity to nitrogen decreases in the order Al–Ga–In, which results in decomposition of the nitrides upon heating below their melting points. In fact, InN is so far only relevant as an admixture to (Al,Ga,In)N mixed crystals because the growth of single crystals is difficult. InN layers were obtained by hydride vapour-phase epitaxy (HVPE), a technique that will be explained below in the context of GaN (Sato & Sato, 1994 ▶), and InN nanowires were obtained from the gas phase in a vapour–liquid–solid (VLS) process (Tang *et al.*, 2004 ▶). The stability of AlN is shown in the *T*–log[*p*] phase diagram in Fig. 4 ▶ where, below atmospheric pressure, AlN(s) is in equilibrium with the gas phase only (sublimation) and at intermediate pressure with the gas phase (containing N_2_ + Al) and the remaining molten Al, and only at high *p* does melting of AlN occur. The calculated triple point here is 2830°C and 17.4 bar. The accurate position of this triple point is still under discussion, and some other references claim high a *p*_N_2__ beyond 100 bar (Ioffe Database; http://www.ioffe.ru/SVA/NSM/Semicond/AlN/thermal.html), but it should be acknowledged that it is almost impossible to measure exact values under such extreme conditions. Experimentally, AlN decomposition starts at a significantly lower *T* than the AlN(s) phase boundary in Fig. 4 ▶ if the material is heated in gases other than N_2_. The current author has obtained a 10% mass loss from a 33 mg AlN sample that was heated in a differential thermal analysis (DTA)/thermogravimetry (TG) apparatus in a helium atmosphere to 2040°C (unpublished results).

The extreme conditions that are required to maintain a solid–liquid equilibrium for AlN make sublimation growth more feasible, and indeed it is typically performed at *T* > 2040°C and *p* ≲ 1 bar (Hartmann *et al.*, 2013 ▶). For GaN the establishment of suitable growth conditions is more difficult, because gallium (in contrast with aluminium) does not evaporate sufficiently for sublimation growth. In fact, Ga is the chemical element with the broadest range of liquid-phase stability under ambient pressure: 29.8 ≤ *T* (°C) ≤ 2203 (*FactSage 6.4 Thermodynamic Databank*; GTT Technologies, 2013 ▶). However, Karpiński *et al.* (1984 ▶) showed that, at high *p* and *T*, nitrogen does dissolve significantly in liquid gallium; a solubility of 1 mol% N_2_ was found at 1500°C and 16 kbar, which proved sufficient to establish melt solution growth of bulk GaN. Other technologies for the growth of bulk GaN rely on chemical transport of the gallium species: from a supercritical ammonia solution, 2 inch GaN crystals can now be grown (Dwilinski *et al.*, 2010 ▶).

**Figure 5:**
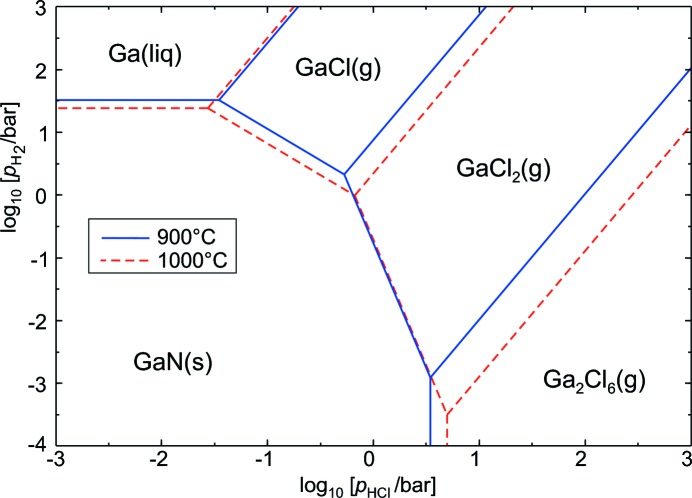
Predominance diagram for the Ga–N–H–Cl system for a prevailing NH_3_ fugacity of 1 bar. Calculated using *FactSage 6.4*.

Hydride vapour-phase epitaxy (HVPE) is an epitaxy process for the deposition of semiconductor layers, including GaN. For this process, metallic gallium reacts at *ca* 850°C with an HCl flow to form gaseous gallium(I) chloride
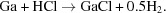
After passing the Ga source, the GaCl/HCl flow (with N_2_ as the carrier gas) reacts with ammonia and gallium nitride is deposited
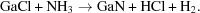
Fig. 5 ▶ shows that, under these process conditions, GaCl gas is in equilibrium with solid GaN and the latter is formed if the HCl fugacity drops distant from the source. Equilibrium (Δ*G* = 0) for the GaN formation reaction given above is reached under ambient pressure at 918°C. Although HVPE is, in principle, a layer growth process, it is also suitable for the production of bulk material, with growth rates of around 100 µm h^−1^ and sample thicknesses of several millimetres possible. However, some drawbacks have to be taken into account: (i) as a side reaction between HCl and NH_3_, large quantities of solid NH_4_Cl are formed that can obstruct the system; (ii) HVPE-grown GaN is bowed, which interferes with the production of planar wafers (Lipski *et al.*, 2012 ▶). Jacobs *et al.* (2010 ▶) showed that, in systems containing graphite, the halogen Cl can be replaced by the pseudo-halogen CN, and gaseous gallium(I) cyanide (GaCN) transports Ga. Crystalline GaN is then deposited either by the thermal decomposition of GaCN or by a reaction that is analogous to that of the common HVPE growth technique given above.

Strite & Morkoç (1992 ▶), and more recently O’Leary *et al.* (2006 ▶) with a deeper insight into electronic properties, reviewed AlN, GaN, InN and their solid solutions, which cover a wide range of *E*_g_: 6.2 ≥ *E*_g_ (eV) ≥ 0.68. Meanwhile, remarkable technological progress has been achieved, and now (Al,Ga,In)N-based devices are the basis for solid-state lighting applications. Because GaN and AlN substrates are still scarce and expensive, homoepitaxy plays no significant role and is still used mainly for basic research (Funato *et al.*, 2012 ▶). Heteroepitaxy is performed on different surfaces of α-Al_2_O_3_ (sapphire), mainly (0001) but also 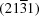 and 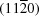. Other useful substrates are several *A* ^III^ *B* ^V^ compounds such as GaAs, SiC polytypes and ZnO. LiAlO_2_ and LiGaO_2_ are interesting alternatives because their epitaxial misfit is much lower compared with *e.g.* sapphire, and large bulk single crystals for substrates up to 2 inch diameter are also available. After epitaxy, the substrates can easily be dissolved in dilute acids, which makes contacting of epitaxial layers from both sides feasible (Liu, 2004 ▶; Veličkov *et al.*, 2008 ▶). Fascinating new possibilities are offered by the integration of GaN in silicon technologies, especially for high-electron-mobility transistors (HEMTs) (Hu *et al.*, 2014 ▶). Even if the lattice mismatch for GaN(0001) on Si(111) is as large as 17%, satisfactory layers can be grown in such ‘GaN-on-Si’ systems by metal–organic chemical vapour deposition (MOCVD) using graded buffer layers (Drechsel *et al.*, 2012 ▶).

Among the other substances in Table 3 ▶, zinc oxide has by far the greatest practical impact nowadays. It is a typical direct wide band gap semiconductor and its properties are reviewed in numerous articles (Look, 2007 ▶; Janotti & Van de Walle, 2009 ▶; Klimm *et al.*, 2011 ▶). As for some other oxide semiconductors, the electronic properties of the ZnO surface are significantly different from the bulk, and can be manipulated by doping or adsorbance layers. The latter effect is used for gas-sensing applications, whereas ZnO ceramics with a small proportion of an additive such as Bi_2_O_3_ or other oxides have an extremely nonlinear resistance resulting from the grain/interlayer/grain boundaries. This nonlinearity is so large that ceramic ‘varistors’ are commercially produced with negligible resistance above and almost infinite resistance below a threshold voltage.

Like most other oxide semiconductors, ZnO is intrinsically *n*-type. Numerous attempts to obtain stable *p*-type conductivity with a technologically adequate hole concentration and mobility have failed so far. This is a severe drawback compared with Al–Ga–In nitride and restricts or even prohibits the manufacture of many devices. If the *n*-type conductivity of ZnO is enhanced, for instance by doping with aluminium, then transparent electrodes, *e.g.* for solar cell applications or flat screen panels, can be produced which are much cheaper than ITO (indium tin oxide) electrodes (Kluth *et al.*, 1999 ▶). Grundmann (2010 ▶) reports an electron concentration of around 10^21^ cm^−3^, a Hall mobility of 47.6 cm^2^ V^−1^ s^−1^ and a resulting specific resistivity of 8.5 × 10^−5^ Ω cm.

## 4. Other oxides   

ZnO is the only semiconducting oxide material presented in Table 3 ▶. As a result of the high electronegativity of oxygen (3.5) compared with the anions of classical semiconductors (S 2.5, P 2.1 and As 2.0), oxides tend to have a comparatively high ionicity and wide *E*_g_. Nitride semiconductors (electronegativity of N = 3.0) are in this respect intermediate between oxides and classical semiconductors.

Many metal oxides are true isolators with a large band gap, such as α-Al_2_O_3_ (corundum). The elements that follow aluminium in group 3 of the periodic system, and some other elements such as tin, lead, bismuth and titanium, have oxides with *E*_g_ values that fall into the range of wide band gap semiconductors. The terms ‘transparent conducting oxide’ (TCO) or ‘transparent semiconducting oxide’ (TSO) are often used for such substances which combine optical transparency with electrical transport properties.

An increasing number of TCO and TSO compounds have been studied during the last decade, and some of them can be found in Table 4 ▶. Ramesh & Schlom (2008 ▶) reviewed the status and prospects of ‘oxide electronics’, which have already yielded some remarkable results with technological relevance. The replacement of SiO_2_ in MOSFET gates by ‘high-κ’ materials, enabling a higher packing density of circuits, is an instructive example. Initially, experiments focused on well known substances such as BaTiO_3_, but failed because the gates degraded. Hubbard & Schlom (1996 ▶) and Schlom & Haeni (2002 ▶) performed a thermodynamic search for metal oxides that are stable in contact with silicon, which has a high oxygen affinity. About one decade after their discovery that hafnium oxide (HfO_2_) belongs to the few compounds which might replace SiO_2_, this high-κ material was introduced into the production of devices. However, thermodynamic equilibria have to be considered not only for the implementation of oxides into silicon electronics; redox equilibria also play a major role in the bulk or layer growth of oxides, more than is typically observed for other anions such as nitride or sulfide.

This difference can be explained mainly by the vast number of different metal oxides that exist, in particular for the subgroup elements, with consequently smaller phase stability fields for each of them. Among the 4776 compounds of the *FactPS* thermodynamic database (*FactSage 6.4*; GTT Technologies, 2013 ▶) can be found *e.g.* for manganese, four oxides, two sulfides and two nitrides; for copper, two oxides, two sulfides and one nitride; and for titanium, 12 oxides, five sulfides and one nitride.

**Figure 6:**
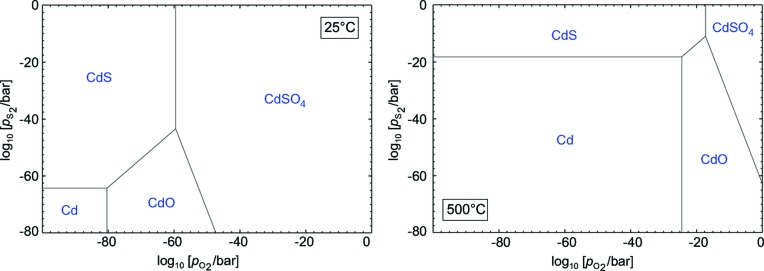
Predominance diagram of the Cd–O–S system for two temperatures.

If metal oxides with oxidation states *m* and *m* + 1 can transform *via* the redox equilibrium
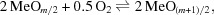
then the Gibbs free energy change of this reaction is proportional to Δ*G* = *RT *ln[*p*_O_2__] if the fugacity of both oxides can be neglected. Plots of *RT* ln[*p*_O_2__] *versus T* are linear and separate predominance fields are observed for subsequent metal oxides (Cahn *et al.*, 1991 ▶; Klimm *et al.*, 2009 ▶). In a similar manner, the behaviour of one or more metals in dependence of several nonmetal fugacities can be calculated and yields predominance diagrams (*T* = constant) with straight phase boundaries. With this type of diagram, Fig. 5 ▶ explains the HVPE process for GaN, and Fig. 6 ▶ shows the equilibria between cadmium, oxygen and sulfur for two different temperatures.

Heterostructures of almost every composition can be grown in modern epitaxial systems and, under sufficiently low *T*, nonequilibrium states can also be produced because they are metastable. However, one should be aware that, over time and especially if *T* increases *e.g.* in an active device, metastable structures might approach equilibrium. This is exactly what happened with BaTiO_3_ MOSFET gates before the introduction of HfO_2_! From Fig. 6 ▶ one reads that CdO and CdS are in equilibrium for a wide *T* range, and consequently heterostructures of the oxide and sulfide are possible. This agrees with experimental results (Li *et al.*, 2009 ▶). On the other hand, for very high fugacities of O_2_ and S_2_, the sulfate CdSO_4_ is a stable intermediate phase between oxide and sulfide. Such an optional intermediate phase should not be forgotten for other heteroepitaxial systems such as nitride and oxide (Shimamura *et al.*, 2005 ▶). The observation of NO_*x*_ bond signatures by photoelectron spectroscopy of oxidized InN surfaces (Eisenhardt *et al.*, 2012 ▶) could hint at the formation of indium nitrate [In(NO_3_)_3_] or nitrite [In(NO_2_)_3_] as an intermediate phase between InN and In_2_O_3_.

Chemical stability considerations also play a major role for the bulk growth of oxide crystals that can be used for substrates. At least ZnO, Ga_2_O_3_, In_2_O_3_ and SnO_2_ have in common that a high (in the case of SnO_2_ not even accessible) melting point exceeding 1800°C is combined with a comparatively high *p*_O_2__, which is necessary to avoid decomposition of the MeO_*x*_ oxide to metal (Me) and oxygen. Certainly for ZnO, a very broad range of methods have been published that allow one to circumvent the stability problem mentioned above, and these methods are more or less suitable for other wide band gap semiconducting oxides as well. These methods include:

(i) growth from solutions, either water- or ammonia-based (often hydrothermal/ammonothermal conditions), or from molten salts (*e.g.* top-seeded solution growth, TSSG);
(ii) growth by physical vapour transport (PVT, sublimation) or chemical vapour transport (CVT);
(iii) growth from the melt, either from cold crucibles (‘skull melting’) or from hot iridium crucibles with a ‘reactive atmosphere’;

and were reviewed elsewhere (Klimm *et al.*, 2011 ▶).

Bulk growth of the most important semiconductors, silicon and gallium arsenide, is exclusively performed from the melt, and ZnO (Schulz *et al.*, 2006 ▶), β-Ga_2_O_3_ (Víllora *et al.*, 2004 ▶; Aida *et al.*, 2008 ▶; Galazka *et al.*, 2010 ▶) and In_2_O_3_ (Galazka *et al.*, 2013 ▶) can also be grown in this way. Tin(IV) oxide (SnO_2_) has not been melt-grown so far; the melting point of this substance is certainly much higher than 1630°C, as given in several references and databases (*FactSage 6.4*; GTT Technologies, 2013 ▶).

**Figure 7:**
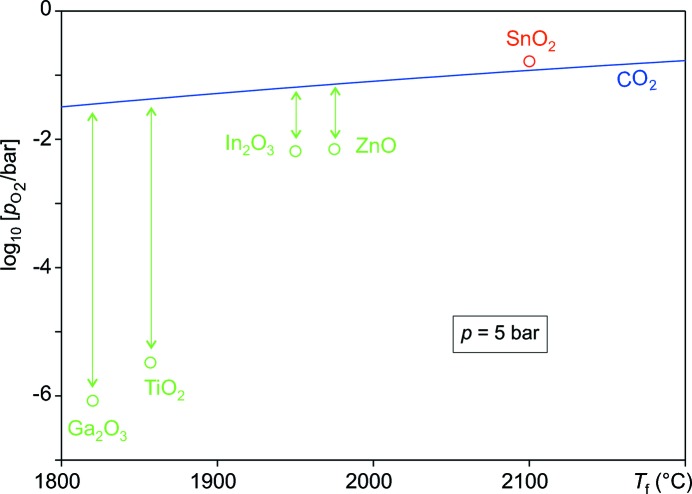
The minimum oxygen fugacities of several transparent conducting oxides at their melting points *T*_f_, compared with the *p*_O_2__(*T*) that results from the thermolysis of carbon dioxide.

Klimm *et al.* (2009 ▶) reported that the partial thermal dissociation of carbon dioxide 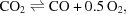
gives the basis for melt crystal growth of oxides in a reactive atmosphere. Pure CO_2_, or (for a somewhat lower oxygen fugacity) CO_2_/CO mixtures, can give such a ‘self-adjusting atmosphere’, where *p*_O_2__(*T*) meets the stability field of the desired oxide for all *T*. For the semiconducting oxides mentioned so far, comparatively large *p*_O_2__(*T*) are required, and Fig. 7 ▶ explains why bulk crystal growth of SnO_2_ from the melt is so difficult: the required oxygen fugacity is so high that it cannot be produced by CO_2_ dissociation. As reported by Galazka *et al.* (2014 ▶), it is not the high melting point itself that is the problem, rather the oxide phase instability becomes an issue if the required oxygen fugacity approaches the *p*_O_2__(*T*) line of CO_2_, and growth is probably impossible this way if it is beyond the line.

For β-Ga_2_O_3_, large bulk crystals (EFG grown ribbons, and float zone and Czochralski boules) are available and epitaxial techniques (MOCVD, MBE) have been developed. Higashiwaki *et al.* (2012 ▶) reported the production of a metal–semiconductor field-effect transistor (MESFET) out of this material, and a high on/off drain current ratio of ∼10 000 was reached. The authors claimed that very high breakdown fields of around 8 MV cm^−1^ should be feasible with β-Ga_2_O_3_, which is almost as good as diamond (10–20 MV cm^−1^) and outperforms both the current high-power material 4H-SiC (2.5 MV cm^−1^) and also GaN (3.3 MV cm^−1^). Monoclinic β-Ga_2_O_3_ is the stable modification between the melting point and room temperature, enabling melt growth of bulk crystals. With epitaxial growth on sapphire (α-Al_2_O_3_) substrates and by alloying, α-(Al,Ga,In)_2_O_3_ layers can be grown which allow band-gap tuning from 3.8 to 8.8 eV (Fujita & Kaneko, 2014 ▶).

Nearly all oxide semiconductors are intrinsically *n*-type, which is a result of the strong localization of holes (if formed by doping or nonstoichiometry) at the oxide ions and impedes the development of devices with *p*–*n* junctions. This is a general problem for all oxide semiconductors and cannot be overcome completely, but some circumstances such as tetrahedral coordination of oxide ions and some degree of covalency can improve *p*-type conductivity (Banerjee & Chattopadhyay, 2005 ▶). Kawazoe *et al.* (1997 ▶) demonstrated that delafossite-type CuAlO_2_ combines an encouraging *p*-type conductivity with transparency to visible light. The carrier density of 1.3 × 10^17^ cm^−3^ and the Hall mobility for holes of 10.4 cm^2^ V^−1^ s^−1^ were explained by a strong hybridization of the oxygen 2*p* orbitals with the 3*d* ^10^ electrons of the Cu^+^ closed shell, leading ultimately to a low hole effective mass. These ideas were the basis of an extended numerical study by Hautier *et al.* (2013 ▶). For 3052 binary and ternary oxides, density functional theory (DFT) computations were performed to identify substances with a low hole effective mass and a large band gap. Some substances which have so far been rarely studied could be promising here: K_2_Sn_2_O_3_, Ca_4_P_2_O, Tl_4_V_2_O_7_, PbTiO_3_, ZrOS, B_6_O and Sb_4_Cl_2_O_5_. It should be noted that the well known good hole mobility of Cu_2_O (which has, however, a small *E*_g_ ≃ 2.1 eV) and CuAlO_2_ could be reproduced too.

**Figure 8:**
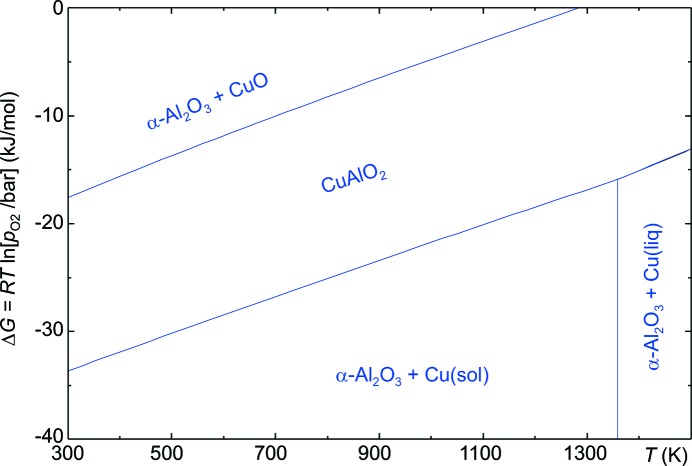
Ellingham predominance diagram of the Cu–Al–O_2_ system with [Cu]:[Al] = 1:1.

Copper exists in most compounds as Cu^2+^, and the untypical low-valency Cu^+^ is directly responsible for the *p*-type conductivity of CuAlO_2_ and Cu_2_O. In a similar manner, SnO_2_ is the ‘normal’ tin oxide and *n*-conducting, whereas potassium stannate(II) (K_2_Sn_2_O_3_; Hautier *et al.*, 2013 ▶), like tin(II) oxide (SnO), shows hole conductivity (Ogo *et al.*, 2008 ▶). It is certainly possible to obtain small quantities of such low-valency oxides, either as epitaxial layers or in the bulk, just by crystallizing them together in an oxygen-poor atmosphere. This was demonstrated by Yoon *et al.* (2013 ▶) with millimetre-sized CuAlO_2_ crystals that could be grown from a Cu_2_O melt flux. Some time ago, Gadalla & White (1964 ▶) showed that, in air, CuAlO_2_ becomes unstable below 1030°C, undergoing partial oxidation to CuO. The stability diagram in Fig. 8 ▶ was calculated using *FactSage 6.4*. It demonstrates that the Cu^I^ compound CuAlO_2_ has a stability field between metallic copper for lower *p*_O_2__ and CuO for higher *p*_O_2__. The calculation of such diagrams requires that thermodynamic data such as *G*(*T*) are available for the intermediate phase, here CuAlO_2_. But even if this is not the case, a coarse approximation can be reached if the relevant phase is simply neglected for the equilibrium calculation. An intermediate phase field, α-Al_2_O_3_ + Cu_2_O, then appears instead at almost the same place; just the upper and lower phase boundaries are shifted *ca* 5 kJ mol^−1^ inwards. This is because the formation energy for Al_2_O_3_ + Cu_2_O 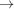 2 CuAlO_2_ is not taken into account. The major contributions to the Gibbs free energy of the system result from the equilibria between the subsequent oxidation states of copper. Hence, it is almost sufficient to have *G*(*T*) data for all relevant element oxides available. One can expect that working in a suitable reactive atmosphere will pave the way to bulk crystal growth conditions where the delafossite phase can be kept thermodynamically stable.

## 5. Summary and conclusions   

The successful story of technological semiconductor applications started in the early 1950s with the first *p*–*n* junctions, which were made inside Czochralski-grown germanium single crystals. For electronic applications, germanium is nowadays replaced almost completely by silicon. Optoelectronics, mainly based on *A* ^III^ *B* ^V^ compounds such as GaAs, opened a new field for semiconductors in the 1970s, but the arsenides and phosphides which could be grown in that time have a narrow band gap, enabling optical emission only from the infrared to the green spectroscopic range. This is sufficient *e.g.* for displays, indicators and optical data transmission, but not for general illumination, as the blue range is missing.

Beginning in the late 1980s, the successful growth of several nitrides, especially the wide band gap semiconductors GaN and AlN on sapphire substrates, widened the accessible wavelength range into the ultraviolet region. White light can now be produced for solid-state lighting, with a positive impact on global energy consumption by replacing incandescent light bulbs with light-emitting diodes.

The substance palette is extended by different polytypes of silicon carbide (SiC) and several oxides, such as ZnO, β-Ga_2_O_3_ and In_2_O_3_. These wide band gap semiconductors are used as substrates for layer deposition of the more classical semiconductors mentioned above, as well as for active devices. Unfortunately, the still unsatisfactory *p*-type conductivity of semiconducting oxides is an issue which significantly hinders the development of devices. Wide band gap semiconductors such as SiC, GaN and β-Ga_2_O_3_ have potential not only for optoelectronics, but also for high-power devices.

For some recently reported organic–inorganic perovskite-type substances such as CH_3_NH_3_PbBr_3_ (Kojima *et al.*, 2009 ▶) and CH_3_NH_3_SnI_3_ (Hao *et al.*, 2014 ▶), band gap tuning is possible by substitution of the halide and/or metal ion. They have been used as absorbers in solar cell structures, enabling power conversion efficiencies greater than 15%. One can hope that further progress is possible here, but it seems too early to include this substance group into this review.

## Figures and Tables

**Table 1:** Semiconductor crystals with the diamond structure

	Diamond	Silicon	Germanium	Grey tin
*a*_0_ (nm)	0.3567	0.5431	0.5658	0.6489
*T* range (°C)	≲1500	<1414	<938	<13
*E*_g_ (eV)	5.48	1.12	0.66	0.08
λ_g_ (µm)	0.226	1.11	1.87	>15

**Table 2:** Some polytypes of SiC (Bechstedt *et al.*, 1997 ▶; Ching *et al.*, 2006 ▶; Tairov & Tsvetkov, 1983 ▶)

	2H (= wurtzite)	4H	15R	6H	3C (= sphalerite)
*a*_0_ (nm)	0.3076	0.30817	0.30817	0.30817	0.43579
*c*_0_/*n*	2.524	2.5198	0.2520	0.2520	0.2517
*E*_g_ (eV)	3.33	3.27	2.986	3.02	2.39

**Table 3:** Semiconductors with the sphalerite (S) or wurtzite (W) structure

	GaN	GaP	GaAs	AlN	ZnO	ZnSe	β-AgI
Type	W	S	S	W	W	S	W
*a*_0_ (nm)	0.319	0.5451	0.5653	0.311	0.3253	0.5668	0.458
*c*_0_ (nm)	0.519	–	–	0.498	0.5213	–	0.7494
*E*_g_ (eV)	3.44	2.26	1.42	6.2	3.3	2.7	2.63
λ_g_ (µm)	0.36	0.59	0.87	0.20	0.38	0.46	0.47

**Table 4:** Some wide band gap oxides

	α-Al_2_O_3_	β-Ga_2_O_3_	In_2_O_3_	SnO_2_	CuAlO_2_
Structure	Corundum	Monoclinic	Bixbyite	Rutile	Delafossite
Space group	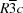	*C*2/*m*	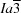	*P*4_2_/*mnm*	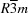
*a*_0_ (nm)	0.51284	1.2214	1.0117	0.47397	0.2857
*b*_0_ (nm)	–	0.30371	–	–	0.2857
*c*_0_ (nm)	–	0.57981	–	0.31877	1.6939
Angle (°)	α = 55.28	β = 103.83	–	–	–
*E*_g_ (eV)	8.3	4.8	3.6	3.6	2.22
